# Use and completion of partograph during labour is associated with a reduced incidence of birth asphyxia: a retrospective study at a peri-urban setting in Ghana

**DOI:** 10.1186/s41043-019-0171-7

**Published:** 2019-05-16

**Authors:** Reindolf Anokye, Enoch Acheampong, Judith Anokye, Amy Budu-Ainooson, Evelyn Amekudzie, Isaac Owusu, Naomi Gyamfi, Agyei Gyimah Akwasi, Wisdom Kwadwo Mprah

**Affiliations:** 1 Centre for Disability and Rehabilitation Studies, Department of Community Health, Kwame Nkrumah University of Science and Technology, Kumasi, Ghana; 2 Kwame Nkrumah University of Science and Technology, Kumasi, Ghana; 3 School of Public Health, Department of Health Education and Promotion, Kwame Nkrumah University of Science and Technology, Kumasi, Ghana; 4 Department of Midwifery, Kwame Nkrumah University of Science and Technology, Kumasi, Ghana

**Keywords:** Disability prevention, Labour, Partograph, Asphyxia, Ghana

## Abstract

**Background:**

Morbidity of birth asphyxia has been estimated to be 42 million disability-adjusted life years. The study sought to assess the impact of the use and completion of partograph during labour on reducing birth asphyxia at the St Anthony’s Hospital, Dzodze, in the Volta Region of Ghana.

**Methods:**

A retrospective study design using a quantitative approach was adopted for the study. A simple random sampling technique was used to select a total of 200 folders of labouring women who were admitted and delivered at St Anthony’s Hospital, Dzodze, between 1st May 2015 and 30th April 2016. A structured checklist, which was developed by using labour and foetal monitoring parameters based on the standards of the World Health Organization partograph usage, was used to review all the 200 existing maternal records.

**Results:**

The findings revealed that partographs were used by midwives at St Anthony’s Hospital with the majority of the maternal folders fully completed. The use and completion of partograph were found to be associated with less non-asphyxiated birth outcomes. Labours which were monitored with partograph were 4.29 times less likely to result in birth asphyxia [AOR (95% CI) 4.29 (1.35–14.81)], and those that were monitored with a completed partograph were 5.3 times less likely to result in birth asphyxia [AOR (95% CI) 5.31 (2.011–16.04)].

**Conclusion:**

Midwives used partographs during labour at St Anthony’s Hospital. The use and completion of partograph were significantly associated with a reduced incidence of birth asphyxia at the hospital. Birth asphyxia could be reduced if partographs are used and completed by midwives during labour in all cases.

## Introduction

Obstructed labour is a significant cause of not only maternal death but also short- and long-term disability [1]. In developing countries, obstructed labour remains one of the primary causes of birth asphyxia [2].

Nearly 1 in every 1000 live births in developed countries, as compared to 5 to 10 in every 1000 live births in developing countries, suffer severe perinatal asphyxia causing death or severe neurological impairment [3]. Lawn et al. [4] highlighted that the disability-adjusted life years (DALYs) denoting the number of years lost due to ill-health, disability, or early death attributed to the burden of morbidity of birth asphyxia is estimated to be 42 million.

The effects of asphyxia in the short term could be multi-organ dysfunction or even death [5] and could result in the development of cerebral palsy; developmental delay; visual, hearing, and intellectual impairment; epilepsy; and learning and behavioural problems in the long term [5, 6]. Birth asphyxia is also concomitant with a wide range of neurodevelopmental and neurological disorders and disability in life [7]. Lawn et al. [4] added that the severe occurrence of birth asphyxia is associated with disorders that extremely blight an individual’s integration into society as an independent adult. Furthermore, it is becoming increasingly apparent that more subtle cognitive and affective deficiencies are primary and permanent consequences of birth asphyxia and are in most need of prevention [8]. According to Gorgos [9], the accompanying effects of birth asphyxia have a resilient and ubiquitous harmful effect on both the quality of life of the child and the caregivers of which include anxiety, depression, and coping problems.

Teenagers with a diagnosis of asphyxia at birth are much more likely to perform poorly in tests of intellectual development and to suffer epilepsy compared to peers with no asphyxia [10]. Cognitive dysfunction may also manifest as problematic school performance, social maladjustment, and behavioural difficulties in adults who had asphyxia at birth [11]. Verbal processing and memory can be negatively affected [12], and vision and hearing can be impaired [13]. Attention deficit hyperactivity disorder, schizophrenia, and autism are neuropsychiatric disorders dominant in survivors of birth asphyxia [14].

To reduce these obstetric complications, the World Health Organization recommends the use of partograph especially in low resourced countries [15]. Partograph is a tool that serves as a first warning system which assists in early decision making on transfer action and ongoing evaluation of the effect of midwifery interventions [16]. It is an inexpensive pictorial, graphical representation of maternal and foetal observations recorded during the active stage of labour [17]. The use of the partograph is recommended for routine monitoring of labour and helps the health care provider in identifying slow progress in labour and aid in timely and appropriate interventions to prevent prolonged labour and obstructed labour [18]. This study sought to evaluate the use and documentation of partograph in labour and the incidence of birth asphyxia at the St Anthony’s Hospital, Dzodze, in the Volta Region of Ghana.

## Methods

### Study site

The study was conducted at the maternity unit of the St Anthony’s Hospital, Dzodze, in the Ketu North district of the Volta Region of Ghana. It is a Catholic Church-owned facility under the Keta-Akatsi Diocese of Ghana. It is a district hospital with a catchment population of about 260,000 people. Referrals are received from Ketu South, Akatsi, Keta, and Ho district. About 25 to 30% of the patients seen in this facility come from the neighbouring Republic of Togo. A total staff strength of 173 includes 20 midwives. The bed capacity of the hospital is 236; out of this, 30 beds serve the maternity unit which consists of antenatal area, delivery area, and lying-in area and the average total number of delivery per month is 120. The hospital was selected because it serves a large catchment population of about 260,000 people. Moreover, it receives referrals from Ketu South, Akatsi, Keta, and Ho district.

### Study design

For this study, a retrospective design was adopted using a quantitative approach. A retrospective study uses existing data that have been recorded for reasons other than research [19]. In healthcare, these are often called “chart reviews” because the data source is the medical record [19].

### Sample and sampling

Folders of all women in labour who were admitted and delivered at St Anthony’s Hospital, Dzodze, between 1 May 2015 and 30 April 2016 were the population used for the study. The 2015/2016 year was randomly selected out of other year groups that were eligible for selection.

The eligibility criteria for selection were folders of labouring women who were admitted and delivered at the facility’s maternity unit between the selected period indicated. Therefore, all folders that were not recorded within this period were excluded from the study. Out of 398 folders, 200 folders of labouring women who were admitted and delivered at the facility’s maternity unit between this period were used as the sample size for the study.

The sample size was calculated using the Yamane formula [20] where 95% confidence level and 0.05 precision levels were used. The calculation of the sample size was based on the Yamane simplified formula for proportions equation:

$$ n=\frac{N}{1+N{\left(\boldsymbol{e}\right)}^2} $$

Therefore, *n* is the sample size, *N* is the population size, and *e* is the level of precision. Overall, 398 folders of labouring women, who were admitted and delivered at the facility’s maternity unit between the period of 1 May 2015 and 30 April 2016, were found. The formula was applied to select the study sample.

A simple random sample was used. In this case, a sample frame was drawn using all the folder numbers of the labouring women who were admitted and delivered within the facility between the period that was considered for the study. The folder numbers were randomly picked until the 200th folder was selected.

### Instrumentation

A structured checklist, which was developed by using labour and foetal monitoring parameters based on the standards of the World Health Organization partograph usage, was used to review all the 200 existing maternal records.

### Data analysis

At the end of the entire data collection process, the data was entered into Statistical Package for Social Sciences (SPSS) version 21.0 and analysed using logistic regression with odds ratio used to present strength of association between risk factors and outcomes. The analysed data was organised into frequency tables and represented on pie charts and tables. The study utilised both descriptive and inferential statistical techniques in data analysis.

### Ethics approval

Ethical clearance was obtained from the committee on Human Research Publications and Ethics (CHRPE) of the Kwame Nkrumah University of Science and Technology.

An introductory letter from the Department of Midwifery of the Kwame Nkrumah University of Science and Technology was obtained to embark on the study. Permission was obtained from the administrator of St Anthony’s Hospital to carry out the study. All information from the client’s folder was treated confidentially. The identity of the study respondents was kept confidential throughout the study. In order to maintain anonymity, participants’ identity was not disclosed for this study. The administrators of the hospital approved the use of the facility for the study, and ethical approval was obtained from them.

## Results

### Demographic data

Table 1 shows an analysis of the distribution of the mother’s demographic data. The results indicate that more than half of the mothers were between 24 and 29 (29%) years as well as between 30 and 35 years (30%).

**Table 1:** Distribution of patients demographic data

Variables	Characteristics	Frequency	Percentage
Age			
	18–23 years	50	25
	24–29 years	58	29
	30–35 years	60	30
	36–41 years	28	14
	Above 41	4	2
Marital status			
	Single	62	31
	Married	134	67
	Divorced/separated	4	2
Parity			
	0	76	38
	1	86	43
	2	14	7
	3	24	12
	4 and above	0	0

The table also shows that the majority (67%) were married while a few (2%) had divorced or were separated.

### Use and documentation of partograph

From Table 2, out of the 200 client’s folders reviewed, 174 representing 87% had partographs in their folders while 26 folders representing 13% had no partographs in them.

**Table 2:** Use and documentation of partograph

Variables	Characteristics	Number	Percentage
Partograph use			
	Yes	174	87
	No	26	13
Partograph completion			
	Yes	139	80
	No	35	20
Cervical dilatation documentation			
	Yes	174	100
	No	0	0
Documentation of descent			
	Yes	158	91
	No	16	9
Documentation of FHR			
	Yes	167	96
	No	7	4
Documentation of amniotic fluid			
	Yes	161	93
	No	13	7
Documentation of foetal head moulding			
	Yes	162	93
	No	12	7
Documentation of uterine contraction			
	Yes	166	95
	No	8	5
Crossing of the action line			
	Yes	149	86
	No	25	14
Mode of delivery	SVD	174	87
	CS	26	13

Out of 174 maternal folders that had partograph, 139 representing 80% were fully completed while 35 representing 20% were not fully completed.

Table 2 further shows that all the 174 folders in which partograph were used had cervical dilatation plotted to standard.

Also, 158 partographs representing 91% had foetal head descent recorded to standard while 16 partographs representing 9% had the descent of the foetal head not recorded at all.

Furthermore, 167 partographs representing 96% had foetal heart rate (FHR) recorded to standard while 7 of the partographs representing 4% had FHR not recorded at all.

From Table 2, 161 partographs representing 93% had amniotic fluid recorded to standard while 13 partographs representing 7% had amniotic fluid not indicated at all. Also, 162 partographs representing 93% had foetal head moulding recorded to standard while 20 partographs representing 7% had foetal head moulding not recorded at all. Table 2 further shows that 166 partographs representing 95% had uterine contraction recorded to standard while eight partographs representing 5% had no uterine contraction recorded at all.

Moreover, 149 partographs representing 86% progressed well without crossing the action line while 25 partographs representing 14.4% crossed the action line.

On the mode of delivery, 174 patients representing 87% had spontaneous vaginal delivery (SVD) while 26 patients representing 13% delivered by Caesarean section (CS).

### Foetal outcome concerning the use and completeness of partograph

**Fig. 1:**
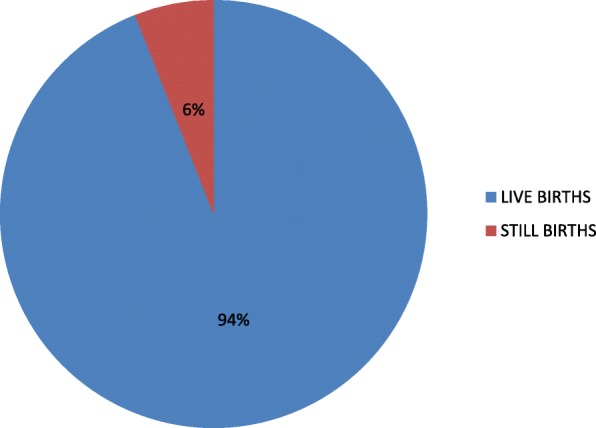
Foetal outcome

Figure 1 shows that 188 babies representing 94% were born alive while 12 babies representing 6% were stillborn.

### Condition of a newborn baby

**Fig. 2:**
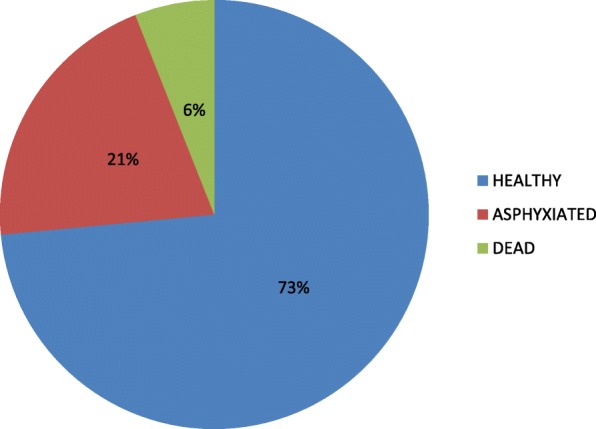
Condition of the baby

Figure 2 shows that out of the 188 live births, 147 babies representing 73% were born with no asphyxia while 41 live births representing 21% were born with different degrees of asphyxia.

### First minute Apgar score

**Fig. 3:**
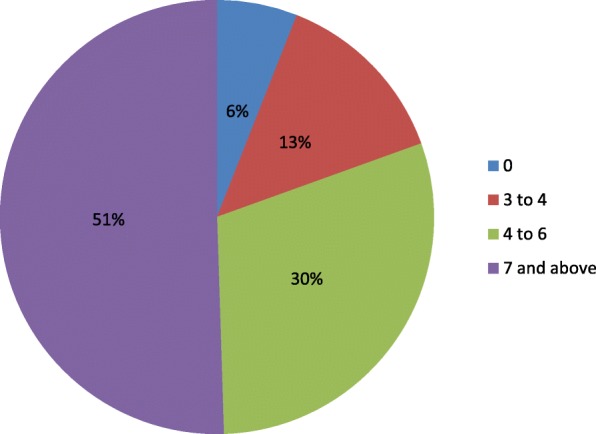
First minute Apgar score

From Fig. 3, babies born with Apgar score of zero at the 1st minute were 12 representing 6%, Apgar score (AS) from 2 to 3 were 27 representing 13%, AS from 4 to 6 were 60 representing 30%, and AS 7 and above were 101 representing 51%.

### Fifth minute Apgar score

**Fig. 4:**
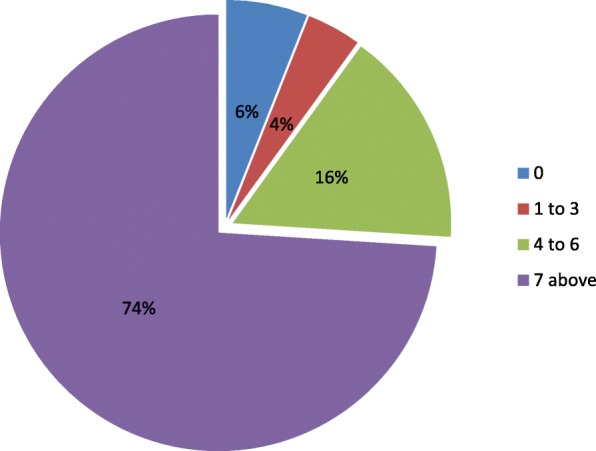
Fifth minute Apgar score

From Fig. 4, babies with Apgar score of zero at the 5th minute were still at 12 representing 6%, Apgar score from 1 to 3 at 5th minute were 8 representing 4%, Apgar score from 4 to 6 were 32 representing 16%, and Apgar scores from 7 and above were 148 representing 74%.

### Foetal outcome and the use of partograph

Table 3 displays a summary of the results of the univariate and multivariate analysis. In both the univariate analysis and the multivariate analysis, the use and documentation completion of partograph had a significant association with a birth outcome such as birth asphyxia (*p* = 0.002) and (*p* = 0.001).

**Table 3:** Association between use and documentation completion of partograph and incidence of asphyxia

Variables	Asphyxia		Univariate		Multivariate^a^	
	Present (*n*)	Absent (*n*)	OR (95% CI)	*P* value	OR (95% CI)	*P* value
Age						
18–23 years	10	40	1.00		1.00	
24–29 years	18	30	2.72	1.317	2.91 (0.14–6.01)	0.312
30–35 years	9	51	1.91 (0.12–18.90)	1.061	1.94 (0.98–8.09)	0.197
36–41 years	3	25	16.5 (3.54–45.49)	0.081	5.14 (1.01–21.8)	0.091
Above 41	1	3	5.12 (0.02–5.21)	1.020	0.62 (0.02–6.00)	0.101
Parity						
0	12	64	1.00		1.00	
1	14	72	1.02 (0.44–2.35)	0.838	1.62 (0.41–4.60)	0.425
2	10	4	1.14 (0.32–4.01)	0.912	2.00 (0.17–21.05)	0.291
3	5	19	1.39 (0.81–1.91)	1.109	1.09 (0.14–1.78)	0.431
4 and above	0	0	1.10 (0.48–2.48)	1.091	0.81 (0.14–3.39)	1.00
Partograph use						
Yes	23	151	5.89 (2.41–23.02)	0.002	4.29 (1.35–14.81)	0.033
No	18	8	1.00		1.00	
Partograph completion						
Yes	9	130	7.28 (4.08–21.17)	< 0.001	5.31 (2.011–16.04)	0.021
No	32	3	1.00		1.00	

*OR* odds ratio, *CI* confidence interval

^a^Mutually adjusted

Multivariate logistic regression analysis indicates that labours which were monitored with partograph were 4.29 times less likely to result in birth asphyxia [AOR (95% CI) 4.29 (1.35–14.81)], and those that were monitored with a completed partograph were 5.3 times less likely to result in birth asphyxia [AOR (95% CI) 5.31 (2.011–16.04)].

## Discussion

Midwives in Ghana received specific training in the use of partograph in 2010 [21]. Knowledge in the use of partograph promotes confidence, reduces the length of labour, and reduces Caesarean section rate and intrapartum stillbirths, but the commitments to its use in providing the desired effects are worrying. Irrespective of how knowledgeable obstetric caregivers are regarding the partograph, its use and documentation of the labour monitoring parameters during labour is often a challenge. The findings revealed that 87% of the folders had indications of partograph use while 13% had no such information on partographs use. This is worrying as partograph use is required in every delivery. A lower proportion of partograph usage was reported by Opoku and Nguah [22] in a study in Ghana where out of 809 deliveries, partograph was used in 54.6% of the deliveries. A slightly higher proportion of usage was reported in a Ugandan study reporting a 69.9% partograph usage during deliveries [23]. However, a deficient proportion of partograph usage was reported in an Ethiopian study, where only 12% of the cases were monitored on a partograph [24].

Contrary to study reports [25] suggesting incomplete documentation of parameters on the partograph by midwives, this study, however, found 80% of the partographs properly filled on all parameters. A study in Tanzania found that only 8.9% of partographs had all parameters completed to standard [26]. A Malawian study also reported that only 10% of folders had partographs filled wholly and correctly [27]. The findings of Opiah et al. [28] suggesting that there is reduced utilisation of the partograph by midwives despite increased knowledge is also in contrast to the findings of this study. The 80% recorded in this study is encouraging and should be commended. Similarly, a study in Ethiopia found that 80% of partographs had key events correctly filled and completed [29]. Nonetheless, the 20% of the partograph that were not wholly filled should be a cause for concern as incomplete documentation of all parameters on the partograph has been associated with late referrals, missing of problems, and perinatal deaths [23, 30, 31]. This suggests the need for ongoing in-service training of midwives on proper usage of the partograph. Proper supervision, monitoring, and supportive follow-ups on midwives together with in-service training will go a long way to improve outcomes for both mothers and foetuses. This has been proven in studies by Fahdhy and Chongsuvivatwong [32] in Indonesia and Ogwang et.al. [23] in Uganda.

The partographs reviewed in this study showed shreds of evidence of documentation of FHR and foetal descent. Ninety-six per cent (96%) and 91% of the partographs had documentation on FHR and foetal descent respectively. However, the parameters that were monitored were not adequately documented. A similar problem was reported in a study in Malawi by Thorsen and Sundby [33]. Monitoring of FHR is strongly associated with outcomes for the foetuses while FHR and descent have been associated with the choice or method of delivery [33]. Other studies have shown that improper monitoring of the FHR may lead to foetal asphyxia and poor Apgar scores [30, 31, 34].

This partly may explain the 21% asphyxiated babies and the 12 (6%) new stillbirths that were recorded in this study. A controlled study reported that before the introduction of partograph, 48 (9.6%) babies needed resuscitation, but this dropped to 21 (4.2%) in those deliveries with proper partographic monitoring. Two (2) fresh stillbirths and seven (7) neonatal deaths were recorded before the introduction of the partograph, but only 2 fresh stillbirths were recorded during the use of partograph and it clearly shows a decrease in perinatal mortality from 3.6 to 0.8%; this shows a significant impact of partograph on neonatal outcomes [35]. The findings of Javed et al. [36] confirm earlier assertions by Bosse et al. [30], Nyamtema et al. [31], and WHO [34]. Monitoring of descent as emphasised earlier has been reported to be strongly associated with the method of delivery. Appropriate decisions on the right method of delivery can be made thereby avoiding maternal and foetal complications [37]. In this study, 87% had spontaneous vaginal delivery (SVD) while 26 patients representing 13% delivered by Caesarean section (CS).

The overall birth outcomes for the foetuses especially were good. This is in contrast to the poor outcomes reported in Malawi by Kitila et al. [27] but consistent with outcomes reported by Javed et al. [36].

Other parameters that were documented on the partograph were foetal head moulding and uterine contraction. Ninety-three per cent (93%) of the partograph had foetal head moulding recorded on them. On the contrary, a study by Bogale and Markos [38] reported that out of the 239 (69.9%) partographs, only 3 (1.3%) of moulding of the foetal head were recorded. Documentation on uterine contraction was 95%. This is somewhat in contrast to the lower percentage of documentation on uterine contractions recorded in a study conducted by Bogale and Markos [38]. The considerable difference in the findings may be attributed to the difference in the sample sizes and the level of utilisation of the partograph based on hospital policies/protocols. A study conducted in eight hospitals in Ecuador reported a massive difference in the utilisation of the partograph among countries and health facilities [39].

This study showed that labours monitored with the partograph were 4.29 times less likely to result in birth asphyxia. This is consistent with that observed in Nigeria, Malawi, Pakistan, and India [40, 27, 36, 41].

### Limitations

The study focused on assessing the association between partograph use and documentation and asphyxia overlooking other maternal and health care system risk factors. Also, 200 folders were assessed, and a more significant number could have been selected if other healthcare facilities were used. However, the findings of the study do not fall short in any way, and the aim of the study was achieved.

## Conclusion

This study confirmed the use of partographs during labour and the relationship that exist between the use of partographs and birth outcomes. It can, therefore, be concluded that the use and completion of partograph were significantly associated with a reduced incidence of birth asphyxia at St Anthony’s Hospital, Dzodze, in the Ketu North district of the Volta Region of Ghana.

## Recommendation

Hospital authorities and the Ministry of Health should ensure that midwives use partographs at the Hospital during labour.

Hospital authorities should ensure that all active labour cases are monitored on a partograph.

Furthermore, a monthly review of clients who delivered at the facility should be regularly done by midwives and hospital authorities.

## Abbreviations

FHR: Foetal heart rate
SPSS: Statistical Package for Social Sciences
